# Binary Political Optimizer for Feature Selection Using Gene Expression Data

**DOI:** 10.1155/2020/8896570

**Published:** 2020-11-29

**Authors:** Ghaith Manita, Ouajdi Korbaa

**Affiliations:** ^1^Laboratory MARS, LR17ES05, ISITCom, University of Sousse, Sousse, Tunisia; ^2^ESEN, University of Manouba, Manouba, Tunisia

## Abstract

DNA Microarray technology is an emergent field, which offers the possibility of obtaining simultaneous estimates of the expression levels of several thousand genes in an organism in a single experiment. One of the most significant challenges in this research field is to select high relevant genes from gene expression data. To address this problem, feature selection is a well-known technique to eliminate unnecessary genes in order to ensure accurate classification results. This paper proposes a binary version of Political Optimizer (PO) to solve feature selection problem using gene expression data. Two transfer functions are used to design a binary PO. The first one is based on Sigmoid function and will be noted as BPO-S, while the second one is based on V-shaped function and will be noted as BPO-V. The proposed methods are evaluated using 9 biological datasets and compared with 8 binary well-known metaheuristics. The comparative results show the prevalent performance of the BPO methods especially BPO-V in comparison with other techniques.

## 1. Introduction

Molecular biology research evolves through the development of technologies used to carry them out. It is not possible to investigate a countless number of genes using conventional strategies. DNA Microarray is a technology that allows researchers to investigate and treat problems that were once considered untraceable. The expression of many genes can be examined in a solitary response rapidly and productively. DNA Microarray technology is enabling the scientific community to understand the fundamental aspects underlying the growth and development of life, as well as to investigate the hereditary reasons for irregularities in the working of the human body.

Therefore, microarray technology remains to this day a useful asset for measuring of gene expression. Beyond the technology itself, the analysis of the data from microarrays is a complex statistical problem. And this is due to the large number of genes and the complexity of biological networks which increase the challenges of understanding and interpreting the resulting mass of data, which often consists of millions of measurements. Hence, extracting relevant biological knowledge from microarray data turns into a hard task due to the curse of dimensionality problem [1].

Generally, gene expression data are often redundant and noisy with large number of genes. In order to reduce the dimensionality of such datasets by selecting the most informative features, Feature Selection (FS) procedure seems to be an essential preprocessing phase before the implementation of machine learning classifiers in order to minimize training times and memory requirements [2].

Feature selection methods are classified into three categories based on the evaluation criteria used: filter, wrapper, and embedded [3]. This categorization depends on the involvement of a learning algorithm in the used approach.

The filter methods (Chi-Square [4], Information Gain [5], Gain Ratio [6], and ReliefF [7]) select a subset of variables by preprocessing the data from a model. The selection process is independent of the classification process. One of the advantages is that it is completely independent of the data model we are trying to build. It proposes a satisfactory subset of variables to explain the structure of the hidden data and that the subset is independent of the chosen learning algorithm. On the contrary, wrapper methods aim to generate representative subsets and evaluate them using a classification algorithm. This evaluation is carried out by calculating a score, e.g., a score of a set will be a compromise between the number of variables eliminated and the success rate of the classification on a test set. Therefore, wrapper methods are more exact than the filter approaches since they consider the relations among the features. Another advantage is its conceptual simplicity; we do not need to understand how induction is affected by the selection of variables, just generate and test. Nevertheless, the computational cost is significantly increased and depends on the used learning algorithm [8]. Finally, embedded methods integrate selection directly into the learning process, and decision trees are the most emblematic illustration. However, we classify in this group all techniques that evaluate the importance of a variable in coherence with the criterion used to evaluate the overall relevance of the model. They are generally known by their reasonable trade-off between efficiency and computing costs [9, 10].

FS is regarded as an NP-complete combinatorial optimization problem [11]. The search space size is strongly dependent to the increase of the number of features in the studied dataset. An exhaustive search for the optimal relevant feature often leads to stagnation in local optima [12]. Therefore, metaheuristic methods are potentially more suitable to deal with this problem because of their ability to find acceptable solutions in reasonable periods of time [13]. The objective function may be the accuracy of the classification or another criterion that could consider the best compromise between the computational burden of attribute extraction and efficiency [14]. Metaheuristics are stochastic approaches and fall into two categories: population-based approaches and single-solution approaches [14, 15]. Generally, they are inspired by nature, social behavior, biological behavior of animals or birds or insects, physical or chemical phenomena, etc.

In the literature, many works were introduced in order to implement stochastic methods to address the FS problem, such as Simulated Annealing (SA) [16], Tabu Search (TS) [17, 18], Genetic Algorithm (GA) [19–22], Particle Swarm Optimization (PSO) [23, 24], Ant Colony Optimization (ACO) [25, 26], Artificial Bee Colony (ABC) [27, 28], and Differential Evolution (DE) [29, 30].

Generally, these traditional methods suffer from a slow convergence rate, and they have a lot number of parameters to be tuned. Hence, a simple and efficient global search technique is needed. For that, during this work, we use the Policy Optimizer (PO) [31] as the main resolution technique since it is a newly introduced metaheuristic which is human behavior-based algorithm. Moreover, as mentioned in [31], PO produces better solutions for dealing with optimization problems than other well-known metaheuristics in the literature. In this paper, a novel binary version is proposed to find the most representative subset of a given dataset. The binary version introduced here is performed using two different transfer functions.

The structure of this paper is as follows: the standard (continuous) version of Political Optimizer (PO) is presented in Section 2. In Section 3, we introduce the binary version of the latter algorithm called BPO. The obtained results and conducted comparisons are reported in Section 4. Finally, the conclusion and several directions for future papers are stated in Section 5.

## 2. Overview of the Political Optimizer (PO)

Political Optimizer is a newly proposed metaheuristic based on human behavior and inspired by the multiphased political process. However, it should be noted that the proposed algorithm is not the first of this kind. In PO, the concept of politics is mapped from a different perspective and unlike the recent politics-inspired algorithms, and this is due to four reasons. First, PO tries to model all the important steps in politics such as party formation, party-ticket/constituency allocation, election campaign and party switching, interparty election, and parliamentary affairs after government formation. Second, PO introduces a novel position updating strategy called recent past-based position updating strategy (RPPUS). This latter represents the learning behavior of politicians from the previous election. Third, each individual solution assumes a double job: a party member and an election candidate. Using this concept, each solution can be updated according to two better solutions: the party leader and the constituency winner. Finally, to improve the results, intermediary solutions needs to cooperate and communicate via a phase named parliamentary affairs.

In PO, each party member is viewed as a candidate solution where its goodwill is considered the position in the search space. Moreover, the evaluation function is computed during the election phase where the number of votes obtained by each member party represents the fitness of the candidate solution.

Political Optimizer (PO) is formed by five main phases as follows: party formation and constituency allocation, election campaign, party switching, interparty election, and parliamentary affairs. It should be mentioned that the first phase (party formation and constituency allocation) is executed only one time to initialize and affect different variables. However, the remaining phases are running in loop, as detailed in Algorithm 1. The used variables in PO are summarized in Table 1.

### 2.1. Party Formation and Constituency Allocation

In the beginning, the population *P* is partitioned in *N* parties, where each party *P*_*i*_ includes *N* members (potential solution). Moreover, each *j*th member is noted as *P*_*i*_^*j*^ and represented by a *d*-dimensional vector, where the value *d* is the number of input variables of the treated problem and *P*_*i*,*k*_^*j*^ is *k*th dimension of *P*_*i*_^*j*^.

**Figure 1:**
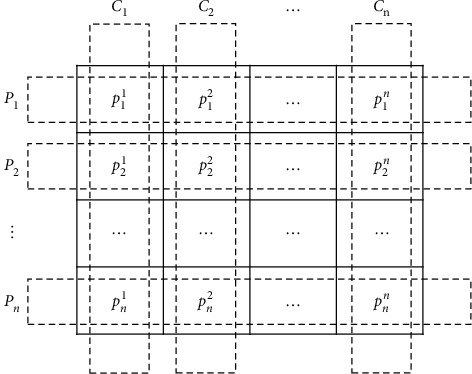
Illustration of the logical division of the population *P* in political parties and constituencies [31].

As mentioned before, each member is considered as an election candidate besides its role as a party member. Hence, *N* constituencies are formed and contain *j*th member of each contesting party. This division is illustrated in Figure 1. Furthermore, the leader of the *i*th party after computing the fitness of all member is noted as *P*_*i*_^*∗*^ and the set of all the party leaders is represented by *P*^*∗*^. On the contrary, after the election, *C*^*∗*^ regroups the winners from all the constituencies named the parliamentarians, where *C*_*j*_^*∗*^ denotes the winner of *j*th constituency.

### 2.2. Election Campaign

During this phase, party members are trying to enhance their chances of being elected by changing their positions according to three aspects. First, they try to learn from previous experience using a novel position updating strategy called recent past-based position updating strategy (RPPUS), as formulated in equations (1) and (2). Second, each party member is trying to update his current position according to the party leader. Finally, candidate positions are updated with reference to the constituency winner:

(1) $$\begin{array}{c} P_{i,k}^{j}\left(t+1\right)=\left\{\begin{array}{cc} m^{*}+r\left(m^{*}-P_{i,k}^{j}\left(t\right)\right), & \mathrm{if}\;P_{i,k}^{j}\left(t-1\right)\leq \;P_{i,k}^{j}\left(t\right)\leq m^{*}\;or\;P_{i,k}^{j}\left(t-1\right)\geq \;P_{i,k}^{j}\left(t\right)\geq m^{*},\\ m^{*}+\left(2r-1\right)\left|m^{*}-P_{i,k}^{j}\left(t\right)\right|, & \mathrm{if}\;P_{i,k}^{j}\left(t-1\right)\leq m^{*}\leq \;P_{i,k}^{j}\left(t\right)\;or\;P_{i,k}^{j}\left(t-1\right)\geq m^{*}\geq \;P_{i,k}^{j}\left(t\right),\\ m^{*}+\left(2r-1\right)\left|m^{*}-P_{i,k}^{j}\left(t-1\right)\right|, & \mathrm{if}\;m^{*}\;\leq P_{i,k}^{j}\left(t-1\right)\leq \;P_{i,k}^{j}\left(t\right)\;or\;m^{*}\geq P_{i,k}^{j}\left(t-1\right)\geq \;P_{i,k}^{j}\left(t\right), \end{array}\right. \end{array}$$

(2) $$\begin{array}{c} P_{i,k}^{j}\left(t+1\right)=\left\{\begin{array}{cc} m^{*}+\left(2r-1\right)\left|m^{*}-P_{i,k}^{j}\left(t\right)\right|, & \;\mathrm{if}\;P_{i,k}^{j}\left(t-1\right)\leq \;P_{i,k}^{j}\left(t\right)\leq m^{*}\;or\;P_{i,k}^{j}\left(t-1\right)\geq \;P_{i,k}^{j}\left(t\right)\geq m^{*},\\ P_{i,k}^{j}\left(t-1\right)+r\left(P_{i,k}^{j}\left(t\right)-P_{i,k}^{j}\left(t-1\right)\right), & \mathrm{if}\;P_{i,k}^{j}\left(t-1\right)\leq m^{*}\leq \;P_{i,k}^{j}\left(t\right)\;or\;P_{i,k}^{j}\left(t-1\right)\geq m^{*}\geq \;P_{i,k}^{j}\left(t\right),\\ m^{*}+\left(2r-1\right)\left|m^{*}-P_{i,k}^{j}\left(t-1\right)\right|, & \mathrm{if}\;m^{*}\;\leq P_{i,k}^{j}\left(t-1\right)\leq \;P_{i,k}^{j}\left(t\right)\;or\;m^{*}\geq P_{i,k}^{j}\left(t-1\right)\geq \;P_{i,k}^{j}\left(t\right). \end{array}\right. \end{array}$$

According to Algorithm 2, which describes the whole process of election campaign, the relationship between current fitness and the previous fitness is the main factor to choose between using equations (1) or (2).

### 2.3. Party Switching

In order to balance between exploration and exploitation, a phase called party switching is started after the election campaign phase. Using an adaptive parameter *λ* named party switching rate, each party member *P*_*i*_^*j*^ can be selected and switched to some randomly chosen party *P*_*r*__._ Hence, it is swapped with the least fit member of the party *P*_*r*_, as presented in Algorithm 3.

### 2.4. Election

This phase aims to evaluate the fitness of all candidates contesting in constituency. After that, the party leaders and constituency winners are updated as follows:

(3) $$\begin{array}{c} q=\arg\min\;f\left(P_{i}^{j}\right),\;\;1\leq i\leq N,\\ C_{j}^{*}=P_{q}^{i},\\ P_{j}^{*}=P_{q}^{i}. \end{array}$$

### 2.5. Parliamentary Affairs

After determining the party leaders and constituency winners (parliamentarians), each parliamentarian aims to improve his performance in order to mimic the interaction and cooperation of the winning candidates to run the government in the postelection phase. This process is presented in Algorithm 4, where each parliamentarian *C*_*j*_^*∗*^  updates its position in relation to randomly chosen parliamentarian *C*_*r*_^*∗*^. It should be noted that the movement is applied only if the performance of *C*_*j*_^*∗*^  is enhanced.

## 3. Binary Political Optimizer (BOP)

As mentioned before, political member's goodwill is considered as a candidate position and moves in the search space towards continuous-valued positions. However, in binary optimization problems, such as feature selection, the search space is modelled as a *n*-dimensional Boolean lattice, and political member's goodwill needs to be represented by binary vectors.

**Figure 2:**
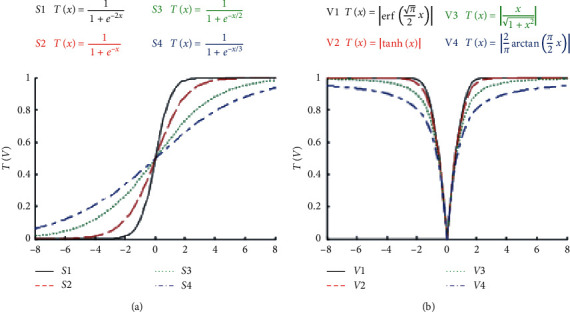
(a) S-shaped and (b) V-shaped family of transfer functions [32].

In order to convert a continuous algorithm to a binary version, we should utilize transfer functions (TF), and it considered as the most efficient and convenient way [32]. Transfer functions are classified into two categories according to their shapes: S-shaped and V-shaped, as illustrated in Figure 2.

In this work, two versions are proposed, based on the transfer function used. In the first one, the political member's goodwill is updated using the Sigmoid function (S-shaped) and called BPO-S. While, in the second one, we used the Hyperbolic Tangent transfer function, called BPO-V.

Without any modification in the previously detailed phases, only two steps are integrated after the continuous computation. The first step is to calculate the probability of changing a position's element to 0 or 1 according to the following equation:

(4) $$\begin{array}{c} P\left(x_{d}^{i}\left(t\right)\right)=\mathrm{TF}\left(x_{d}^{i}\left(t\right)\right), \end{array}$$

where TF is the used transfer function that could be Sigmoid (equation (5)) or Hyperbolic Tangent (equation (6)) and *x*_*d*_^*i*^(*t*)  is the *i*th political member in the *d*th in the iteration *t*:

(5) $$\begin{array}{c} \mathrm{TF}\left(x\right)=\frac{1}{1+e^{-x}}, \end{array}$$

(6) $$\begin{array}{c} \mathrm{TF}\left(x\right)=\left|\tanh\left(x\right)\right|. \end{array}$$

In the second step, the probability computed by equation (4) is then inserted in equation (7) in order to convert continuous value of each member position to 0 or 1:

(7) $$\begin{array}{c} x_{d}^{i}\left(t\right)=\left\{\begin{array}{cc} 1, & \text{if }P\left(x_{d}^{i}\left(t\right)\right)\geq \;\mathrm{rand,}\\ 0, & \mathrm{otherwise,} \end{array}\right. \end{array}$$

where rand is a uniform random number between 0 and 1.

**Figure 3:**
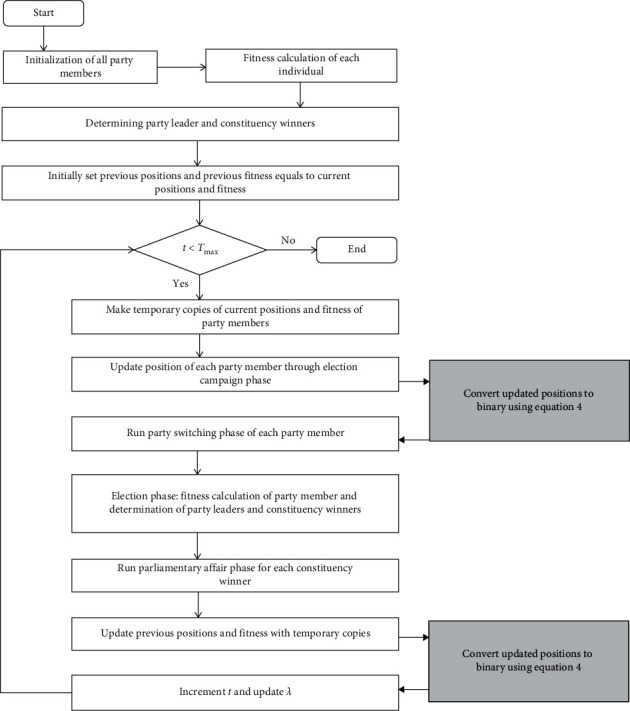
Flowchart of the proposed algorithm.

The flowchart of the proposed binary algorithm is presented in Figure 3.

### 3.1. Binary Political Optimizer Applied for Feature Selection

In this section, we exploited the proposed BPO in feature selection for classification problems. As mentioned before, the feature selection problem is an NP-hard combinatorial binary optimization problem. For a feature vector sized *N*, the different feature combinations would be 2*N* which increase exponentially the number of possible solutions where an exhaustive search is probably not practical. Therefore, we used the proposed BPO in order to find an acceptable solution with reasonable execution time. The main objective is to maximize the classification accuracy and minimize the number of selected features. The used fitness function is presented in the following equation [33]:

(8) $$\begin{array}{c} ↑F=\mathrm{Acc}+\;\omega \;\left(1-\;\frac{sf}{nf}\right), \end{array}$$

where Acc is the classification accuracy given a chosen classifier, *ω* is the weight factor which is a value between 0 and 1, *sf* is the length of selected feature subset, and *nf* is the total number of features. In this study, we set *ω* to 0.5 for all the experiments in the next section. For the classifier, we chose to use *k*-Nearest Neighbor (*k*-NN) to compute the accuracy of selected subset. Moreover, to ensure the robustness of the obtained results, every used dataset is divided randomly into two different parts: training and testing set, according to 10-fold crossvalidation method.

## 4. Experimental Results

In this section, all experiments were repeated for 100 independent times to obtain statistically meaningful results. Furthermore, each algorithm was implemented using MATLAB R2020a and was run on an Intel Core i7 machine, 2.6 GHz CPU, and 16 GB of RAM.

### 4.1. Dataset

In this study, nine benchmark biological datasets are used to assess the performance of the proposed approach [34–44].  Table 2 outlines the datasets used in this work.

### 4.2. Parameter Settings

To evaluate the proposed model, several experiments were conducted to compare the BPO algorithm with seven different metaheuristic optimization algorithms: Binary Particle Swarm Optimization (BPSO) [45], Binary Genetic Algorithm (BGA) [46], Binary Bat Algorithm (BBA) [47], Binary Differential Evolution (BDE) [48], Binary Grey Wolf Optimizer (BGWO) [49], Binary Atom Search Algorithm (BASO) [50], Binary Harris Hawks Optimizer (BHHO) [51], and Binary Tree Growth Algorithm (BTGA) [52]. The parameters settings for all metaheuristic optimization algorithms are shown in Table 3.

### 4.3. Results and Discussion

In this section, we start to evaluate statically the performance of the two proposed version of BPO compared to other algorithms. Therefore, four different statistical measures are used to start the first step of evaluation. These measurements were the worst fitness value, the best fitness value, the mean fitness value (avg), and standard deviation (std).  Table 4 outlines the obtained results using these measures where the best ones are highlighted in bold text. From the table, we assess the superiority of proposed algorithms, especially BPO-V, compared to others binary version of well-known algorithms. However, BPO-V and BPO-S can be described as unstable methods in most cases. This fact can be explained by the complexity of position update strategy adopted by PO. Furthermore, it can be observed that BASO is the most competitive algorithm with the two version of BPO. From these findings, it can be concluded that BPO-V is better than BPO-S, BGA, BGWO, BBA, BHHO, BDE, BASO, BPSO, and BTGA in extracting the most relevant feature of the tested datasets with the aim to maximize the classification performance and minimization of the number of selected features. This deduction was confirmed by applying a Wilcoxon Ranked Signed Test to the proposed algorithms compared in pairs with the other algorithms. This test is performed with a statistical significance value *α* = 0.05. In Tables 5 and 6, the sign “+” in the winner lines designates that the null hypothesis is rejected and the proposed algorithms (BPO-S or BPO-V) statistically outperform in pairs the other ones with 95% significance level (*α* = 0.05). In case of inferiority, the sign “−” is used. From these tables, we can reaffirm in first place the superiority of BPO-S and BPO-V. Moreover, as mentioned before, the BASO algorithm is the most concurrent algorithm.

**Figure 4:**
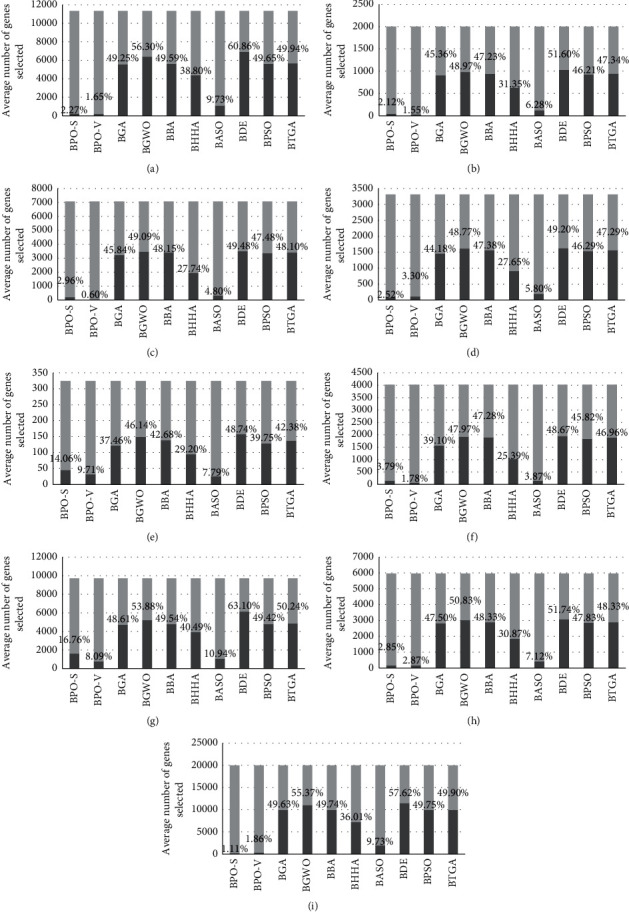
Average number of genes (features) selected for each of the 9 datasets (numbers on the bars indicate the percentage of selected genes).

In the second step, to confirm this superiority, BPO-S and BPO-V are evaluated in terms of accuracy and average number of selected features. From Table 7, it can be concluded that BPO-S and BPO-V outperform in an inescapable way the other algorithms regarding the number of selected features. Hence, Figure 4 is drawn to better visualize the obtained results. One more time, BASO showed the most competitive behavior. On the contrary, Table 8 outlines the comparative results in term of accuracy, where it can be seen that BPO-V is the best algorithm. Therefore, the proposed algorithms strongly reduce the number of selected features without losing important information to deal with the problem treated by the dataset.

**Figure 5:**
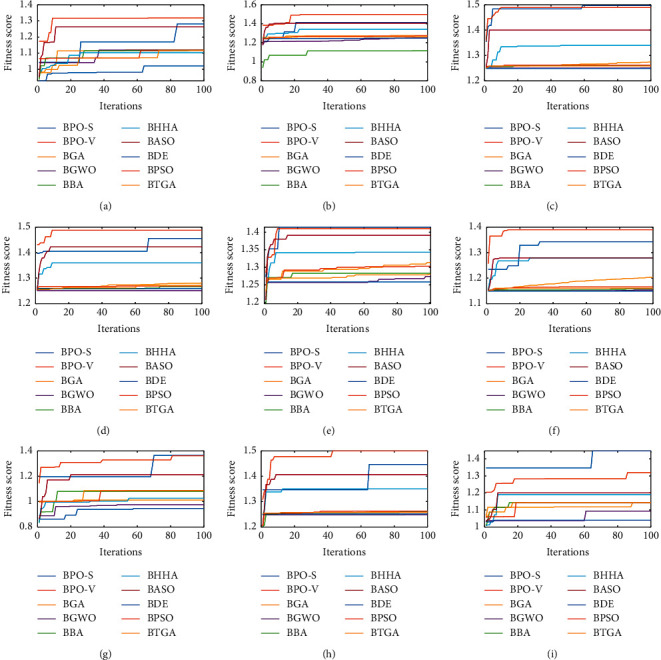
Convergence curves of the proposed approaches compared to 8 metaheuristics for each of the 9 datasets.

At the end of this evaluation, we compare BPO-V and BPO-S in terms of execution time and convergence. Regarding convergence speed and best fitness score obtained, Figure 5 shows that BPO-V also excels in this point. Generally, after 20 iterations, it reaches its optimum solution. On the contrary, despite the good results of BPO-S in terms of fitness score, this algorithm arrives at its best performance late, generally after 50 iterations. In the second term and which concerns the execution time, BPO-V and BPO-S showed poor results according to Table 9. This fact can be explained by the complexity of the algorithm proposed in [31] and its large number of functions to execute and large number of conditions to verify.

## 5. Conclusions

In this paper, we proposed two versions of binary PO algorithm and applied to feature selection problem on gene expression data. To assess the robustness of our work, we used 9 standard datasets characterized by their huge dimensionality. Obtained results are compared to 8 binary versions of well-known metaheuristics. Experimental results prove the excellence performance of proposed algorithm. The results are evaluated using different indicators assessing convergence, reduction size, accuracy, performance (fitness score), and runtime. In future work, BPO could be hybridized with other metaheuristic algorithms as well as another classifier instead of KNN such as SVM.

## Figures and Tables

**Algorithm 1:**
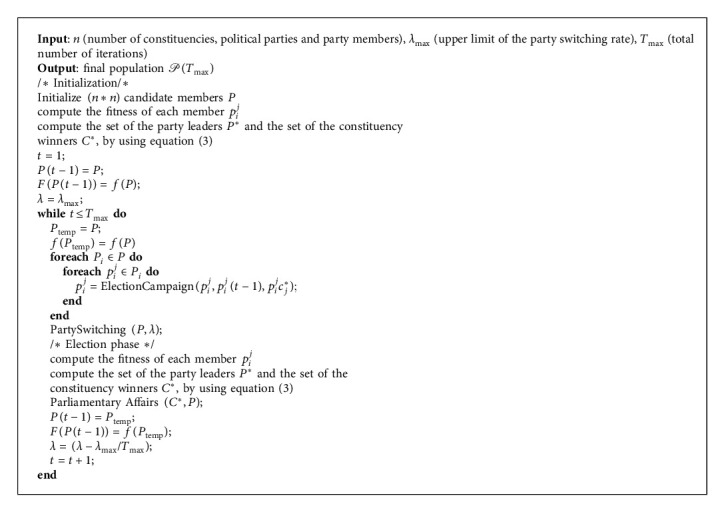
Pseudocode of PO.

**Algorithm 2:**
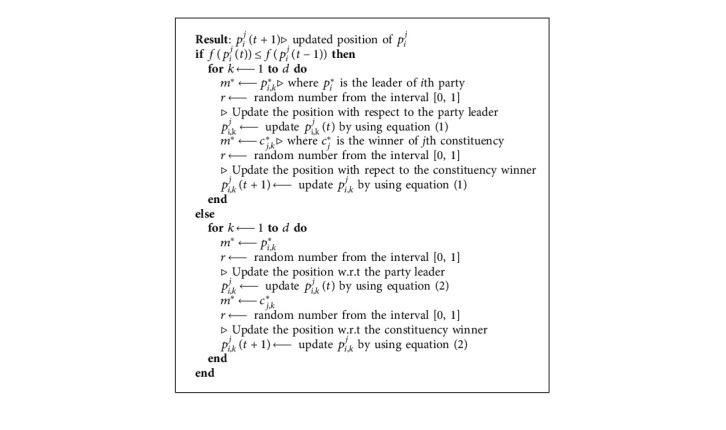
ElectionCampaign (*p*_*i*_^*j*^, *p*_*i*_^*j*^(*t* − 1), *p*_*i*_^*j*^, *c*_*j*_^*∗*^).

**Algorithm 3:**
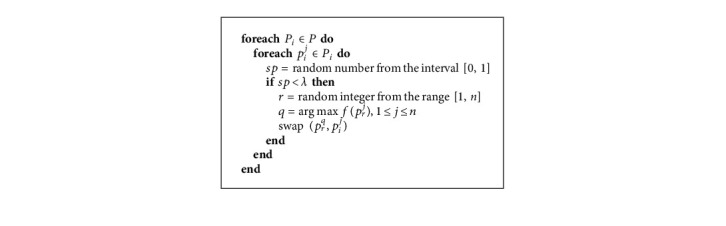
PartySwitching (*P*, *λ*).

**Algorithm 4:**
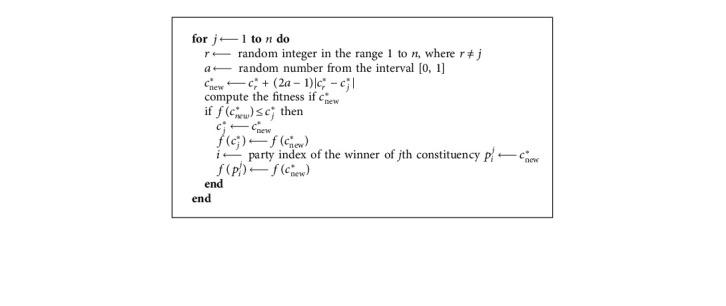
PartySwitching (parliamentary affairs (*C*^*∗*^, *P*)).

**Table 1:** List of the used variables.

Variable	Description
*P*	Set of all political parties (whole population)
*P*_*i*_	*i*th political party
*P*_*i*_^*j*^	*j*th member of *i*th party
*P*_*i*,*k*_^*j*^	*k*th dimension of *j*th member of *i*th political party
*C*	Set of all constituencies
*C*_*j*_	*j*th constituency
*P*_*i*_^*∗*^	Leader of *i*th political party
*C*_*j*_^*∗*^	Winner of *j*th constituency
*λ*	Party switching rate
*N*	Number of parties, constituencies, and members in each party
*T*_max_	Total number of iterations

**Table 2:** Details of datasets.

Dataset	No. of instances	No. of features	No. of classes	Type
CLL_SUB_111 [34]	111	11340	3	Continuous, multiclass
Colon [35]	62	2000	2	Discrete, binary
Leukemia [36]	72	7070	2	Discrete, binary
Lung [37]	203	3312	5	Continuous, multiclass
Lung_discrete [38]	73	325	7	Discrete, multiclass
Lymphoma [39]	96	4026	9	Discrete, multiclass
nci9 [40, 41]	60	9712	9	Discrete, multiclass
Prostate_GE [42, 43]	102	5966	2	Continuous, binary
SMK_CAN_187 [44]	187	19993	2	Continuous, binary

**Table 3:** Parameter settings for all used algorithms.

Algorithm	Parameter	Value
BPO	Parties (number of political parties)	5
	Lambda (max limit of party switching rate)	1

BPSO	c1 (cognitive factor)	2
	c2 (social factor)	2
	Vmax (maximum velocity)	6
	Wmax (maximum bound on inertia weight)	0.9
	Wmin (minimum bound on inertia weight)	0.4

BGWO	a	2

BBA	Goma	1
	Alpha	1
	Zigma	1
	Beta	1
	frequencyMin	20
	frequencyMax	50

BDE	CrossRate	0.9

BTGA	N1 (number of trees in first group)	3
	N2 (number of trees in second group)	5
	N4 (number of trees in fourth group)	3
	Tree reduction rate	0.8
	Parameter controls nearest tree	0.5

BHHO	Beta (levywalk)	1.5

BASO	Alpha (depth weight)	50
	Beta (multiplier weight)	0.2
	Vmax (maximum velocity)	6

BGA	crossoverRate	0.9
	mutationRate	0.1

All of them	SearchAgent(Bats, wolfs, particles,…)	30
	Maximum iterations	100

**Table 4:** Experimental result of the fitness function of the proposed algorithms compared to eight metaheuristics.

Dataset		BPO-S	BPO-V	BGA	BGWO	BBA	BHHO	BASO	BDE	BPSO	BTGA
CLL_SUB_111	Best	1.409	1.4217	1.2065	1.1769	1.2059	1.2547	1.4237	1.1254	1.2053	1.208
	Avg	1.2509	1.3254	1.1287	1.0926	1.1239	1.1433	1.2922	1.0498	1.1254	1.119
	Worst	1.0681	1.2528	1.0707	1.0368	1.0701	1.0894	1.2164	0.9753	1.0684	1.0685
	std	0.0698	0.0347	0.0262	0.0288	0.0264	0.0317	0.037	0.0268	0.0282	0.0296

Colon	Best	1.4995	1.4998	1.284	1.2632	1.2715	1.3807	1.4888	1.2637	1.275	1.2712
	Avg	1.4302	1.4922	1.2732	1.2551	1.2639	1.3433	1.4888	1.242	1.269	1.2633
	Worst	1.3308	1.433	1.2637	1.237	1.257	1.3093	1.45	1.2043	1.2635	1.258
	std	0.0527	0.0163	0.0036	0.0052	0.0026	0.0165	0.0086	0.0144	0.0025	0.0024

Leukemia	Best	1.4999	1.4999	1.276	1.2612	1.2627	1.3848	1.4914	1.2576	1.267	1.2637
	Avg	1.458	1.497	1.2708	1.2546	1.2593	1.3613	1.476	1.2526	1.2626	1.2595
	Worst	1.2772	1.4792	1.2649	1.2494	1.2567	1.3397	1.4628	1.2477	1.2597	1.2566
	std	0.0433	0.0039	0.002	0.0022	0.0012	0.0102	0.0064	0.0023	0.0012	0.0015

Lung	Best	1.4967	1.4953	1.2876	1.2634	1.2674	1.3818	1.4857	1.2655	1.2736	1.2714
	Avg	1.4491	1.4758	1.2791	1.2561	1.2631	1.361	1.471	1.254	1.2685	1.2635
	Worst	1.394	1.4502	1.2716	1.2462	1.2597	1.341	1.4576	1.2446	1.2654	1.2601
	std	0.0228	0.0101	0.003	0.0034	0.0016	0.0091	0.0064	0.0036	0.0019	0.002

Lung_discrete	Best	1.4892	1.4938	1.3292	1.2954	1.3062	1.3815	1.48	1.2862	1.3231	1.3062
	Avg	1.3954	1.4257	1.3127	1.2693	1.2866	1.354	1.4611	1.2563	1.3012	1.2881
	Worst	1.2631	1.3409	1.2954	1.2308	1.2754	1.32	1.4292	1.2108	1.2877	1.2754
	std	0.0562	0.0281	0.0072	0.0136	0.0063	0.0124	0.0096	0.0137	0.0067	0.0064

Lymphoma	Best	1.422	1.4389	1.2083	1.161	1.1616	1.2821	1.4268	1.1574	1.1696	1.1644
	Avg	1.3632	1.388	1.1992	1.1549	1.1583	1.2678	1.3785	1.1514	1.1656	1.1599
	Worst	1.1578	1.3489	1.1909	1.1501	1.1563	1.2532	1.3636	1.1465	1.1624	1.1567
	std	0.0358	0.0105	0.0036	0.0023	0.0012	0.006	0.0104	0.0022	0.0016	0.0016

nci9	Best	1.3296	1.3267	1.1735	1.1629	1.1726	1.2093	1.3127	1.0949	1.1728	1.1663
	Avg	1.0929	1.1854	1.0703	1.0431	1.0681	1.0809	1.217	0.9878	1.0679	1.053
	Worst	0.9786	1.0738	1.0061	0.975	1.0022	0.993	1.1225	0.9168	1.0025	1.0006
	std	0.0729	0.0509	0.043	0.0401	0.0403	0.0428	0.0393	0.0375	0.0394	0.0422

Prostate_GE	Best	1.4972	1.4999	1.2674	1.2583	1.2614	1.3822	1.4828	1.2572	1.2649	1.2622
	Avg	1.4272	1.4751	1.2625	1.2458	1.2583	1.3452	1.4644	1.2413	1.2609	1.2584
	Worst	1.2551	1.4378	1.258	1.2182	1.2557	1.3098	1.446	1.2059	1.2575	1.255
	std	0.0452	0.0204	0.0019	0.0114	0.0014	0.0151	0.0089	0.0135	0.0015	0.0016

SMK_CAN_187	Best	1.3883	1.3916	1.1738	1.1665	1.1959	1.261	1.3986	1.1425	1.1969	1.1976
	Avg	1.2885	1.3294	1.1351	1.107	1.134	1.1691	1.3057	1.0722	1.1278	1.1305
	Worst	1.1754	1.276	1.1118	1.0616	1.089	0.0261	1.2642	1.0299	1.089	1.0889
	std	0.043	0.0231	0.017	0.0226	0.0203	0.0261	0.0261	0.0225	0.0197	0.024

**Table 5:** Pairwise statistical comparison of the BPO-S algorithm with other algorithms using the Wilcoxon signed-rank test (*α* = 0.05).

Dataset		BPO-S	BPO-V	BGA	BGWO	BBA	BHHA	BASO	BDE	BPSO	BTGA
CLL_SUB_111	*p*-value	—	1.786*E* − 04	1.827*E* − 04	1.827*E* − 04	1.786*E* − 04	1.827*E* − 04	1.827*E* − 04	1.827*E* − 04	1.827*E* − 04	1.827*E* − 04
	Winner	—	−	+	+	+	+	−	+	+	+

Colon	*p*-value	—	1.575*E* − 04	1.766*E* − 04	1.766*E* − 04	1.746*E* − 04	1.776*E* − 04	1.776*E* − 04	1.766*E* − 04	1.776*E* − 04	1.756*E* − 04
	Winner	—	−	+	+	+	+	−	+	+	+

Leukemia	*p*-value	—	1.817*E* − 04	1.817*E* − 04	1.817*E* − 04	1.806*E* − 04	1.827*E* − 04	1.827*E* − 04	1.817*E* − 04	1.806*E* − 04	1.817*E* − 04
	Winner	—	−	+	+	+	+	+	+	+	+

Lung	*p*-value	—	1.827*E* − 04	1.827*E* − 04	1.827*E* − 04	1.827*E* − 04	1.827*E* − 04	1.827*E* − 04	1.817*E* − 04	1.796*E* − 04	1.817*E* − 04
	Winner	—	−	+	+	+	+	−	+	+	+

Lung_discrete	*p*-value	—	1.817*E* − 04	1.806*E* − 04	1.817*E* − 04	1.766*E* − 04	1.817*E* − 04	1.776*E* − 04	1.786*E* − 04	1.806*E* − 04	1.806*E* − 04
	Winner	—	−	+	+	+	+	−	+	+	+

Lymphoma	*p*-value	—	1.827*E* − 04	1.817*E* − 04	1.817*E* − 04	1.817*E* − 04	1.817*E* − 04	1.000*E* + 00	1.817*E* − 04	1.817*E* − 04	1.796*E* − 04
	Winner	—	−	+	+	+	+	=	+	+	+

nci9	*p*-value	—	3.600*E* − 03	1.405*E* − 04	2.730*E* − 04	3.447*E* − 04	1.817*E* − 04	1.932*E* − 03	1.706*E* − 03	8.501*E* − 04	5.708*E* − 04
	Winner	—	−	+	+	+	+	+	+	+	+

Prostate_GE	*p*-value	—	4.600*E* − 03	1.806*E* − 04	1.806*E* − 04	1.817*E* − 04	7.650*E* − 04	1.004*E* − 03	1.817*E* − 04	1.806*E* − 04	1.806*E* − 04
	Winner	—	−	+	+	+	+	+	+	+	+

SMK_CAN_187	*p*-value	—	2.100*E* − 02	1.827*E* − 04	1.827*E* − 04	1.827*E* − 04	1.827*E* − 04	9.097*E* − 01	1.827*E* − 04	1.827*E* − 04	1.817*E* − 04
	Winner	—	−	+	+	+	+	=	+	+	+

**Table 6:** Pairwise statistical comparison of the BPO-V algorithm with other algorithms using the Wilcoxon signed-rank test (*α* = 0.05).

Dataset		BPO-S	BPO-V	BGA	BGWO	BBA	BHHA	BASO	BDE	BPSO	BTGA
CLL_SUB_111	*p*-value	1.786*E* − 04	—	1.786*E* − 04	1.786*E* − 04	1.786*E* − 04	1.786*E* − 04	5.354*E* − 02	1.786*E* − 04	1.786*E* − 04	1.786*E* − 04
	Winner	+	—	+	+	+	+	=	+	+	+

Colon	*p*-value	1.575*E* − 04	—	1.612*E* − 04	1.612*E* − 04	1.593*E* − 04	1.621*E* − 04	3.954*E* − 04	1.612*E* − 04	1.621*E* − 04	1.602*E* − 04
	Winner	+	—	+	+	+	+	+	+	+	+

Leukemia	*p*-value	1.817*E* − 04	—	1.806*E* − 04	1.806*E* − 04	1.796*E* − 04	1.817*E* − 04	2.821*E* − 04	1.806*E* − 04	1.796*E* − 04	1.806*E* − 04
	Winner	+	—	+	+	+	+	+	+	+	+

Lung	*p*-value	1.827*E* − 04	—	1.827*E* − 04	1.827*E* − 04	1.827*E* − 04	1.827*E* − 04	4.274*E* − 01	1.817*E* − 04	1.796*E* − 04	1.817*E* − 04
	Winner	+	—	+	+	+	+	=	+	+	+

Lung_discrete	*p*-value	1.817*E* − 04	—	1.796*E* − 04	1.806*E* − 04	1.756*E* − 04	1.806*E* − 04	1.238*E* − 02	1.776*E* − 04	1.796*E* − 04	1.796*E* − 04
	Winner	+	—	+	+	+	+	=	+	+	+

Lymphoma	*p*-value	1.827*E* − 04	—	1.817*E* − 04	1.817*E* − 04	1.817*E* − 04	1.817*E* − 04	1.133*E* − 02	1.817*E* − 04	1.817*E* − 04	1.796*E* − 04
	Winner	+	—	+	+	+	+	=	+	+	+

nci9	*p*-value	3.600*E* − 03	—	1.827*E* − 04	3.298*E* − 04	5.828*E* − 04	1.827*E* − 04	1.827*E* − 04	1.827*E* − 04	1.827*E* − 04	5.828*E* − 04
	Winner	+	—	+	+	+	+	+	+	+	+

Prostate_GE	*p*-value	4.600*E* − 03	—	1.817*E* − 04	1.817*E* − 04	1.827*E* − 04	1.827*E* − 04	7.337*E* − 01	1.827*E* − 04	1.817*E* − 04	1.817*E* − 04
	Winner	+	—	+	+	+	+	=	+	+	+

SMK_CAN_187	*p*-value	2.100*E* − 02	—	1.806*E* − 04	1.806*E* − 04	1.806*E* − 04	1.806*E* − 04	3.108*E* − 02	1.806*E* − 04	1.806*E* − 04	1.796*E* − 04
	Winner	+	—	+	+	+	+	+	+	+	+

**Table 7:** The average number of selected features of the proposed algorithms compared to eight metaheuristics.

Dataset	BPO-S	BPO-V	BGA	BGWO	BBA	BHHO	BASO	BDE	BPSO	BTGA
CLL_SUB_111	257.3	187.62	5585.41	6384.14	5623.11	4399.46	1103.75	6901.91	5630.84	5662.63
Colon	42.34	31.03	907.2	979.4	944.51	626.94	125.54	1032.07	924.18	946.87
Leukemia	209.45	42.16	3240.73	3470.59	3404.03	1961.36	339.51	3498.41	3356.92	3400.45
Lung	83.61	109.25	1463.4	1615.38	1569.27	915.67	192.23	1629.41	1533.21	1566.4
Lung_discrete	45.69	31.56	121.74	149.94	138.7	94.9	25.31	158.4	129.2	137.73
Lymphoma	152.41	71.52	1574.2	1931.39	1903.45	1022.39	155.81	1959.52	1844.89	1890.76
nci9	1627.87	785.56	4720.81	5232.5	4811.22	3932.79	1062.24	6128.74	4799.88	4879.49
Prostate_GE	170.27	171.27	2833.86	3032.71	2883.41	1841.66	424.93	3086.76	2853.46	2883.27
SMK_CAN_187	222.56	371.68	9922.05	11069.31	9945.36	7199.64	1945.67	11519.65	9945.69	9977.48

**Table 8:** The average accuracy of the proposed algorithms compared to eight metaheuristics.

Dataset/time (s)	BPOV1	BPOV2	BGA	BGWO	BBA	BHHO	BASO	BDE	BPSO	BTGA
CLL_SUB_111	0.6864	0.6909	0.6818	0.5909	0.4545	0.5455	0.5000	0.6818	0.5000	0.5909
Colon	0.8667	0.8500	0.8333	0.8333	0.7500	0.7500	0.8167	0.7500	0.7500	0.7500
Leukemia	0.7714	0.9286	0.9286	0.8571	0.8571	0.8571	0.7143	0.7857	0.8571	0.9286
Lung	0.9200	0.9250	0.9150	0.9150	0.9150	0.9000	0.8750	0.9250	0.9150	0.9200
lung_discrete	0.8214	0.8571	0.7857	0.7857	0.8571	0.7143	0.6429	0.8571	0.7857	0.7143
Lymphoma	0.8263	0.8947	0.8947	0.7895	0.8421	0.8947	0.7895	0.8421	0.8947	0.8947
nci9	0.4417	0.5167	0.3333	0.3333	0.5000	0.5000	0.2500	0.5000	0.4167	0.5000
Prostate_GE	0.8650	0.9500	0.9000	0.9500	0.8500	0.9000	0.8500	0.7500	0.9500	0.9000
SMK_CAN_187	0.8949	0.8516	0.6216	0.8108	0.8649	0.7297	0.7027	0.5946	0.6757	0.7568

**Table 9:** The average execution time of the proposed algorithms compared to eight metaheuristics.

Dataset/time (s)	BPO-S	BPO-V	BGA	BGWO	BBA	BHHO	BASO	BDE	BPSO	BTGA
CLL_SUB_111	211.5163	166.0972	156.3265	156.1411	166.7361	269.3532	245.7208	172.91	235.5515	160.3811
Colon	103.2834	110.327	94.1061	96.418	94.6258	164.3746	105.777	89.9312	123.4293	93.9638
Leukemia	140.8718	117.0147	113.6496	127.0532	127.4182	195.2044	170.5102	115.6307	137.6324	110.3661
Lung	168.1855	135.1064	130.194	132.89	125.6319	227.7096	157.0562	139.6336	126.6119	116.7391
lung_discrete	111.4208	108.97	94.692	90.6665	82.4157	178.5376	101.8686	93.4482	80.0484	78.4514
Lymphoma	139.1397	117.5715	135.3171	109.7464	114.0983	202.2584	147.4745	116.1915	103.0365	100.1243
nci9	163.066	127.4642	141.5162	138.7996	137.8389	226.385	236.8451	130.9642	128.7595	116.3961
Prostate_GE	161.4066	131.6549	125.0905	130.5117	130.1564	211.7674	188.3833	134.1632	119.473	114.9097
SMK_CAN_187	519.7175	351.3269	253.5977	342.1177	332.1609	564.8705	423.7244	368.1337	342.0833	289.6829

## Data Availability

The data used to support the findings of the study are available at http://featureselection.asu.edu/datasets.php.
